# Relationship between ethanol consumption and *TBL2* rs17145738 on LDL-C concentration in Japanese adults: a four season 3-day weighed diet record study

**DOI:** 10.1186/s40795-019-0315-6

**Published:** 2019-12-19

**Authors:** S. Akimoto, C. Goto, K. Kuriki

**Affiliations:** 1 Laboratory of Public Health, Graduate School of Integrated Pharmaceutical and Nutritional Sciences, University of Shizuoka, Shizuoka, Japan; 2 Department of Health and Nutrition, Nagoya Bunri University, Inazawa, Japan; 3 Laboratory of Public Health, School of Food and Nutritional Sciences, University of Shizuoka, 52-1 Yada, Suruga-ku, Shizuoka, 422-8526 Japan

**Keywords:** Seasonal variation, Repeated measures data, Missing value, Multiple imputation, Mixed-effect model, Weighed dietary record, Food frequency questionnaire, Single nucleotide polymorphism, Interaction, Serum lipid

## Abstract

**Background:**

LDL cholesterol (LDL-C) concentration is modified by dietary and genetic factors; however, little is known about the details of this relationship. Our aim was to investigate the associations taking into account dietary assessment methods, seasonal effects and missing values.

**Methods:**

Study subjects completed food frequency questionnaires (FFQ) and supplied 3-day weighed dietary records (WDRs) and blood samples in four seasons. Approximately 660,000 single nucleotide polymorphisms (SNPs) were measured. Candidate SNPs related to LDL-C concentration were systematically selected. Multiple imputation was applied for missing values. A total of 312 repeated measures data were used for analyses. After adjusting for season and subjects as fixed and random effects, effects of nutrient intake and SNPs on LDL-C concentration were assessed according to three dietary assessment methods: the FFQ and first and four season 3-day WDRs (4 s-3d WDRs).

**Results:**

For LDL-C concentration, ethanol consumption derived from all three dietary assessment methods was consistently associated (*P* < 0.09 for all). Positive and negative relationships were consistently shown with rs651007 and rs1160985 in the first and four seasons; but the latter remained after adjusting for total dietary fiber intake derived from the FFQ and 4 s-3d WDRs (*P* < 0.05, excepting the first 3-day WDRs). rs599839 was negatively associated after cholesterol intakes derived from the first and 4 s-3d WDRs were considered (*P* < 0.05 and 0.07, respectively). Each rs17145738 and ethanol consumption based on the 4 s-3d WDRs was related to LDL-C concentration (*P* < 0.05). Seasonal variations of LDL-C concentration were observed only in summer.

**Conclusions:**

In contrast to nutrient intake, ethanol consumption was shown to be comprehensively related to LDL-C concentration, regardless of dietary assessment methods. Taking into account seasonal effects, critical relationships with LDL-C concentration for some SNPs, after adjustment for specific nutrients, were revealed. Our findings can be used to help to interpret the relationships between dietary and genetic factors on LDL-C concentration in large-scale epidemiological studies.

(10/10 keywords)

## Background

Globally, approximately 17.9 million deaths in 2016 were estimated to be caused by cardiovascular disease (CVD) [1], making effective and practical strategies to reduce CVD morbidity a priority. Higher concentration of LDL cholesterol (LDL-C) is an important risk factor for developing CVD and an important target for treatment [2, 3]. The highest risk for hypercholesterolemia has been reported to occur in winter [4, 5]. For this reason, the season in which health examinations are undergone might result in under- or over-estimation of LDL-C concentration [5].

In addition to increased consumption of total dietary fiber (TDF), there is a recommendation in the guidelines to reduce dietary cholesterol intake in order to lower LDL-C concentration [2, 3]. However, a negative association with LDL-C concentration has been inconsistent for moderate alcohol consumption, yet [6], and studies to date have failed to show that such reduction in dietary intake is effective. Methodological issues concerning inaccurate dietary assessment method have been proposed as one of the factors for these unexpected findings [7, 8]. In clinical trials, weighed dietary record (WDR) is used to assess actual ingestion of foods and nutrients during research periods, as reference data, in studies with relatively few subjects [9]. In contrast, in many large epidemiological studies, a food frequency questionnaire (FFQ) is often administered to large numbers of subjects in order to estimate habitual dietary intake of foods and nutrients, because FFQs demonstrate less burden for both subjects and researchers. Some FFQs have been developed that take seasonal variation into account [10, 11].

In genome-wide association studies (GWAS) of European people, associations between 39 loci and LDL-C concentration have been identified [12]. Although 22 loci have been found among Japanese people [13], only 4 of these loci were reported to be the same as in the European populations. Some single nucleotide polymorphisms (SNPs) in the lipid metabolism-related genes on LDL-C concentration have been shown to interact with dietary fat intake [14, 15]. Until now, there have been insufficient investigations into the degree of relationship between dietary and genetic factors for disease. Moreover, little is known about the relationship among genetic factors when considering seasonal variations of LDL-C concentration and dietary factors.

Even if individual WDRs were collected for a long period, missing values are excluded in the analysis, resulting in selection bias. If it is the case that the study subjects were appropriately extracted from their population, the bias becomes a critical issue in generalizing results. Multiple imputation (MI) is an established approach to deal with missing values, but much care must be taken [16, 17]. In the present study, we used MI to clarify relationships between dietary and genetic factors on LDL-C concentration. In addition, seasonal effects and dietary assessment methods were taken into account, using the FFQ and 3-day WDRs in each of two study designs - a cross-sectional study and a seasonal variation study.

## Methods

### Study design

A total of 91 participants were recruited from January 2013 to March 2014 as part of the Sakura Diet Study. This study is part of the Japan Multi-Institutional Collaborative Cohort (J-MICC) Study in the Shizuoka-Sakuragaoka area, and has been described previously [18, 19]. This investigation was conducted according to the Declaration of Helsinki human subject principles, and the study protocol was approved by the ethics committee of the University of Shizuoka (No. 24–24). In brief, a representative sample of citizens living in Shizuoka city, Japan was recruited and asked by to supply both a 3-day WDR and a blood sample in each of the four seasons. Written informed consent was obtained from each participant by trained medical staff. At the beginning of the study (i.e., the first season, winter), subjects filled out a FFQ and a lifestyle questionnaire with questions about their medical history. Exclusion criteria for the current study included the following: 1) subjects with dyslipidemia, diabetes, angina pectoris or cerebral stroke; and 2) subjects who did not provide > = 8 h’ fasting blood samples in any seasons.

There were two study designs: 1) a cross-sectional design, using data based on the FFQ, 3-day WDRs and a serum lipid profile collected in the first season; and 2) a seasonal variation design, using data derived from 3-day WDRs and a serum lipid profiles collected in each season. Three datasets were prepared: i) ‘complete cases (CC)’ composed of subjects with no missing values in any of the four seasons; ii) ‘observed subjects (OS)’, which included subjects who provided data in each season, even if some values were missing; and iii) a MI dataset calculated by means of applying the MI procedure to the OS dataset.

### Dietary assessments and lifestyle factors

Initially, 26 nutrient intakes were estimated using the FFQ, with 47 food items over the past 1 year [10, 20, 21]. Subsequently, using three-nonconsecutive-day WDRs, subjects were asked to record their eaten foods and drinks on two weekdays and one weekend day in each season. All 12-day WDRs were systematically reviewed by two trained registered dieticians. According to *Standard Tables of Food Composition in Japan, 2015* (seventh revised edition) and other items [22–25], individual representative intake of foods and nutrients was given using an average value of WDRs for 3 days in each season. Food items not found in these sources were replaced with similar items in order to convert nutrients, with the advice of two expert registered dieticians (i.e., nutritional epidemiologists), who were involved with the J-MICC study. Nutrients were highlighted include fat, cholesterol, TDF and ethanol. Energy-adjusted intake was used for all analyses, except ethanol.

Using a self-administered lifestyle questionnaire, alcohol consumption, including consumption of six types of alcoholic beverages (sake, shochu (distilled spirit), chuuhai, beer, whiskey, and wine) was assessed [26]. In brief, ethanol consumption (g/day) of current alcohol consumers was estimated based on the frequency and amount of each type of alcoholic beverage consumed over the past year, with reference to 180 ml of sake (one “go”), 633 ml of beer (one bottle), 108 ml of shochu containing 25% ethanol, etc., as 23 g of ethanol. Smoking status was categorized as either current smoker (which included a small number of ex-smokers) or non-smoker. Physical activity was assessed by self-report and included questions about the number of hours of daily activity and leisure-time exercise which was then converted into metabolic equivalent task hours (METs•hour/day) [27].

### Biochemical and anthropometric measurements

Concentrations of LDL-C, total and HDL cholesterol and triglyceride levels were measured by an automatic biochemical analyzer in the SRL clinical laboratory (CAP ISO 15189). LDL-C concentration was calculated by Friedewald equation [28], or applied measurement values of LDL-C in the case of subjects with triglyceride levels > = 4.49 mmol/L. Height and weight were measured within 0.1 cm and 0.1 kg respectively, after which body mass index (BMI) was calculated.

### Genotyping and quality control

DNA was extracted by a QIAcube (Tokyo, Japan) with a Qiagen DNA extraction kit (QIAamp DNA Blood Mini Kit). All 659,253 tag SNPs were genotyped with the Japonica array (Toshiba, Tokyo, Japan), which covered 96.9% of common SNPs (minor allele frequency > 5%) and 67.2% of low-frequency SNPs (0.5% < minor allele frequency < =5%) [29]. In addition, this array was reported to provide better imputation performance for Japanese individuals than other SNP arrays with reference to 1KJPN and the International 1000 genomes projects panel. After systematically reviewing the GWAS findings on LDL-C concentration in East Asians (key terms; GWAS, LDL-C and lipids, and eligible populations; Japanese, Korean and Chinese), a total of 39 SNPs related to LDL-C concentration were selected as candidates with reference to their coding genes and minor allele frequencies (Additional file 1) [13, 30–35]. Characterized SNPs found in American-European people were excluded because of being higher LDL-C concentrations than in East Asian populations [36, 37]. Of the 39 candidate SNPs, 10 were measured with Japonica array: *PSRC1* rs599839; *HMGCR* rs12654264; *TIMD4* rs6882076; *TBL2* rs17145738; rs651007; intron between *ABO* and *LCNIP2* rs579459; *LDLR* rs7258950 and rs2738446; *TOMM40* rs1160985; *APOC1* rs445925. The remaining SNPs were not covered by the array chip because they are minor allele frequencies. After quality control checks, three additional SNPs were excluded from the analyses for the following reasons; 1) the call rate of rs2738446 was < 100%; 2) rs7258950 was not in Hardy-Weinberg equilibrium (*P* < 0.05); and 3) rs579459 was in linkage disequilibrium with rs651007 (*r* = 1.0).

### Handling of missing values

Utilizing the ‘mice’ package, MI was conducted using chained equations for missing values [38]. Missing values were assumed to be missing at random, and 50 and 100 imputed datasets (m) and 40 iterations were executed. The assumption was assessed with a density plot [38]. Without collinearity in the models, an imputed dataset was created; subsequently, a number of iterations were counted using the ‘norm2’ package. To investigate the convergence of MI, a trace plot was drawn [38], and results from m = 50 (data not shown) and 100 were compared. Sensitivity analysis was applied to assess the robustness of the results under a missing not at random assumption [38].

### Statistical analysis

All analyses were performed using R software, version 3.4 (R Project for Statistical Computing), and a *P* < 0.05 was considered statistically significant. The continuous and categorical variables were represented as mean ± SD and by adding the numbers and percentages. Non-normally distributed variables such as LDL-C concentration, were log-transformed. To avoid the value of zero, 1 g/day of ethanol consumption was assigned before the log-transformation, and no effect was observed after this assignment. Using the ‘lmerTest’ package, a mixed-effect model was applied to assess seasonal variation, and the two variables, season and subject, were used as a fixed effect (‘1 to 4’ for ‘winter to autumn’) and a random intercept (‘a serial number’), respectively [39]. Dunnett’s test was used for post hoc test with reference to winter. For quality control of genotyping, Hardy-Weinberg equilibrium was examined using chi-square tests, and linkage disequilibrium was evaluated with Pearson’s correlation coefficients.

In the cross-sectional study, a multiple linear regression was applied to LDL-C concentration to detect any independent relationships with nutrient intake and SNPs (homozygous for the major allele/other genotypes = 0/1). Variables used as conventional confounding factors included age, BMI, physical activity, ethanol consumption, and total energy intake (which was excepted in case of SNPs) as continuous variables; and sex (men/women = 0/1) and smoking status (non−/smokers = 0/1) as categorical variables. In the seasonal variation study, using the ‘lmerTest’ package, a mixed-effect model was applied, taking into account each effect of season and subject. Subsequently, interactions between nutrient intake and SNPs were calculated when the *P* < 0.20 was found in the OS dataset of the seasonal variation study.

## Results

In the cross-sectional study, the CC and OS datasets were composed of 51 and 73 subjects, respectively. According to mixed-effect models, seasonal variations were shown in LDL-C, total and HDL cholesterol (*P* < 0.05 for all) (Table 1), and the values were lower in summer. The percentage of missing values was ~ 14%, and characteristics of the subjects were slightly different between the two datasets (data not shown).

**Table 1:** Characteristics of study subjects in four seasons (*n* = 78)

	Winter^a^	Spring^a^	Summer^a^	Autumn^a^	*P*^b^
	Mean or n SD or (%)	Mean SD	Mean SD	Mean SD	
Men	51 (65.4%)				
Smoker^c^	13 (16.7%)				
Age (years)	43.5 ± 9.6				
Physical activity (METs・hour/day)	9.0± 2.0				
BMI (kg/m^2^)	22.4 ± 1.2	22.4 ± 1.2	22.3 ± 1.2	22.4 ± 1.2	0.30
Triglyceride (mmol/L)	0.90 ± 1.72	0.93 ± 1.78	0.92± 1.68	0.91 ± 1.69	0.61
Total cholesterol (mmol/L)	5.35 ± 0.91	5.12 ± 0.76**	5.12 ± 0.86**	5.11 ± 0.83	**<0.01**
HDL cholesterol (mmol/L)	1.67 ± 1.26	1.56 ± 1.25***	1.62 ± 1.26**	1.60 ± 1.26**	**<0.001**
LDL cholesterol (mmol/L)	3.06 ± 1.30	2.95 ± 1.25	2.89 ± 1.31**	2.95 ± 1.26	**0.02**
3-day WDRs					
Energy intake (MJ/day)	8.2 ± 1.7	8.0 ± 2.3	7.9 ± 1.9	8.1 ± 2.0	0.48
Protein (g/MJ)	8.6 ± 1.2	8.6 ± 1.2	8.5 ± 1.1	8.6 ± 1.2	1.00
Fat (g/MJ)	7.6 ± 1.5	7.8 ± 1.5	7.5 ± 1.4	7.7 ± 1.4	0.35
SFA (g/MJ)	2.2 ± 0.6	2.2 ± 0.5	2.2 ± 0.5	2.2 ± 0.5	0.92
MUFA (g/MJ)	2.8 ± 0.6	2.9 ± 0.7	2.8 ± 0.6	2.9 ± 0.6	0.37
PUFA (g/MJ)	1.7 ± 1.3	1.7 ± 1.3	1.6 ± 1.3	1.7 ± 1.3	0.09
Cholesterol (mg/MJ)	35.0 ± 1.4	37.8 ± 1.4	37.9 ± 1.4	38.9 ± 1.4	0.13
Carbohydrate (g/MJ)	31.8 ± 3.6	31.4 ± 3.9	31.9 ± 3.7	32.0 ± 3.8	0.55
TDF (g/MJ)	1.7 ± 1.4	1.7 ± 1.4	1.6 ± 1.4	1.8 ± 1.4	0.33
Ethanol (g/day)	2.2 ± 2.5	2.1 ± 2.8	2.3 ± 2.7	1.7 ± 2.3	0.58
FFQ					
Energy intake (MJ/day)	7.2 ± 1.4				
Protein (g/MJ)	7.3 ± 1.2				
Fat (g/MJ)	6.2 ± 1.6				
SFA (g/MJ)	1.5 ± 0.4				
MUFA (g/MJ)	2.3 ± 0.7				
PUFA (g/MJ)	1.7 ± 1.3				
Cholesterol (mg/MJ)	32.6 ± 1.3				
Carbohydrate (g/MJ)	34.3 ± 3.5				
TDF (g/MJ)	1.4 ± 1.3				
Ethanol (g/day)	5.1 ± 3.9				

*Abbreviations*: *3-day WDRs* 3-day weighed dietary records, *SFA* saturated fatty acid, *MUFA* monounsaturated fatty acid, *PUFA* polyunsaturated fatty acid, *TDF* total dietary fiber, *FFQ* food frequency questionnaire. The following variables were log-transformed to improve normality: physical activity; BMI; triglyceride; HDL cholesterol; LDL cholesterol; protein; PUFA; dietary cholesterol; TDF; ethanol consumption. *P* <0.05 is shown as bold. *P <0.05, **P <0.01, ***P <0.001 compared with winter using Dunnett's test. ^a^The mean and SD were estimated from the subjects without missing values in each season. ^b^Mixed-effect models were applied for analyzing seasonal variation with seasons (1, winter; 2, spring; 3, summer; 4, autumn) as a fixed effect and subject ID as a random intercept, and the analyses were performed using as much data as possible. ^c^Ex-smokers were defined as subjects who quit smoking more than 2 years previously and were included as smokers because of being a small number of them.

In all datasets and dietary assessment methods, negative relationships were found between LDL-C concentration and ethanol consumption (*P* = < 0.01 to 0.09 for all, with the exception of *P* = 0.12) (Table 2). The values of LDL-C concentration were found to be lower in summer (*P* < 0.05 for all). In the cross-sectional study, using a multiple regression model, LDL-C concentration was found to have a negative association with TDF intake only in the CC dataset (*P* = 0.02). In the seasonal variation study with a mixed-effect model, positive relationships were found between LDL-C concentration and dietary cholesterol intake (*P* = 0.02 and 0.04 in the CC and OS datasets, respectively), but this relationship was diminished in the MI dataset. Lower LDL-C concentrations were shown in spring and summer (*P* < 0.05 for both). Density and trace plots show adequate validity of the missing at random assumption and appropriate convergence of MI (data not shown). Sensitivity analysis shows that the results have adequate robustness under a missing not at random assumption (data not shown).

**Table 2:** Associations between nutrient intake and LDL-C concentration according to three dietary assessment methods

	Complete case dataset^a^			Observed subject dataset^b^			Multiple imputation dataset (m = 100)^c^		
	β	SE	*P*	β	SE	*P*	β	SE	*P*
Cross-sectional study									
FFQ (one year)^d^									
Number of observations (person · season)		51			73			78	
Fat (g/MJ)	7.9 × E-3	1.1 × E-2	0.48	9.9 × E-3	9.9 × E-3	0.32	1.1 × E-2	9.7 × E-3	0.27
Cholesterol (mg/MJ)	2.3 × E-2	1.6 × E-1	0.89	1.3 × E-2	1.3 × E-1	0.92	2.9 × E-2	1.3 × E-1	0.82
TDF (g/MJ)	-3.2 × E-2	1.9 × E-1	0.87	1.3 × E-2	1.5 × E-1	0.93	3.1 × E-2	1.5 × E-1	0.84
Ethanol (g/day)	-7.3 × E-2	2.6 × E-2	**0.008**	-3.8 × E-2	2.1 × E-2	0.07	-3.6 × E-2	2.1 × E-2	0.09
First season, 3-day WDR^d^									
Number of observations (person · season)		51			72			78	
Fat (g/MJ)	3.4 × E-4	1.2 × E-2	0.98	-6.3 × E-3	8.9 × E-3	0.48	-6.9 × E-3	8.6 × E-3	0.42
Cholesterol (mg/MJ)	6.0 × E-2	1.3 × E-1	0.64	-1.2 × E-2	9.1 × E-2	0.89	-9.0 × E-3	8.7 × E-2	0.92
TDF (g/MJ)	-2.9 × E-1	1.2 × E-1	**0.02**	-1.2 × E-1	9.0 × E-2	0.20	-1.0 × E-1	8.9 × E-2	0.26
Ethanol (g/day)	-7.9 × E-2	3.1 × E-2	**0.02**	-6.3 × E-2	2.4 × E-2	**0.01**	-5.8 × E-2	2.4 × E-2	**0.02**
Seasonal variation research									
Four season, 3-day WDR^e^									
Number of observations (person · season)		204			262			312	
Fat (g/MJ)	-5.3 × E-4	3.8 × E-3	0.89	-1.0 × E-3	3.2 × E-3	0.76	-1.0 × E-3	4.2 × E-3	0.81
Season - Winter	Reference			Reference			Reference		
- Spring	-1.4 × E-2	9.2 × E-3	0.13	-1.6 × E-2	7.9 × E-3	**0.04**	-1.6 × E-2	1.1 × E-2	0.14
- Summer	-2.8 × E-2	9.1 × E-3	**0.003**	-2.5 × E-2	7.8 × E-3	**0.002**	-2.2 × E-2	1.0 × E-2	**0.04**
- Autumn	-1.4 × E-2	9.1 × E-3	0.13	-1.5 × E-2	8.2 × E-3	0.08	-1.1 × E-2	1.1 × E-2	0.34
Cholesterol (mg/MJ)	7.2 × E-2	3.1 × E-2	**0.02**	5.3 × E-2	2.6 × E-2	**0.04**	5.0 × E-2	3.8 × E-2	0.19
Season - Winter	Reference			Reference			Reference		
- Spring	-1.7 × E-2	9.0 × E-3	0.06	-1.8 × E-2	7.7 × E-3	**0.02**	-1.8 × E-2	1.0 × E-2	0.09
- Summer	-3.0 × E-2	9.0 × E-3	**0.001**	-2.7 × E-2	7.8 × E-3	**<0.001**	-2.4 × E-2	1.0 × E-2	**0.02**
- Autumn	-1.8 × E-2	9.0 × E-3	**0.05**	-1.7 × E-2	8.2 × E-3	**0.04**	-1.3 × E-2	1.1 × E-2	0.24
TDF (g/MJ)	-7.1 × E-3	3.3 × E-2	0.83	-2.2 × E-2	2.9 × E-2	0.46	-2.7 × E-2	4.3 × E-2	0.52
Season - Winter	Reference			Reference			Reference		
- Spring	-1.4 × E-2	9.1 × E-3	0.12	-1.7 × E-2	7.8 × E-3	**0.03**	-1.6 × E-2	1.1 × E-2	0.12
- Summer	-2.8 × E-2	9.2 × E-3	**0.003**	-2.5 × E-2	7.9 × E-3	**0.002**	-2.3 × E-2	1.1 × E-2	**0.03**
- Autumn	-1.4 × E-2	9.0 × E-3	0.12	-1.5 × E-2	8.2 × E-3	0.07	-1.1 × E-2	1.1 × E-2	0.34
Ethanol (g/day)	-2.1 × E-2	1.3 × E-2	0.12	-1.9 × E-2	1.0 × E-2	0.07	-2.7 × E-2	1.5 × E-2	0.08
Season - winter	Reference			Reference			Reference		
- Spring	-1.5 × E-2	8.9 × E-3	0.11	-1.7 × E-2	7.8 × E-3	**0.03**	-1.6 × E-2	1.1 × E-2	0.13
- Summer	-2.8 × E-2	8.9 × E-3	**0.002**	-2.4 × E-2	7.7 × E-3	**0.002**	-2.2 × E-2	1.1 × E-2	**0.04**
- Autumn	-1.4 × E-2	9.0 × E-3	0.12	-1.5 × E-2	8.2 × E-3	0.07	-1.1 × E-2	1.1 × E-2	0.34

*Abbreviations*: *LDL-C* LDL cholesterol, *FFQ* food frequency questionnaire, *3-day WDR* 3-day weighed dietary records, *TDF* total dietary fiber, *m* number of imputations. The following variables were log-transformed to improve the normality: LDL-C concentration; dietary cholesterol; TDF; BMI; physical activity; ethanol consumption. *P* <0.05 was shown as bold. ^a^Complete case dataset was composed of subjects without missing values on four seasons. ^b^Observed subjects dataset was used for the analysis of all of values collected in each season even if there were missing values. ^c^Multiple imputation datasets were calculated by means of applying observed subjects dataset to the MI procedure. ^d^Multiple regression models were applied for analyzing the associations between nutrient intake and LDL-C concentration with adjustment for age, sex (0, men; 1, women), BMI, physical activity, ethanol consumption, smoking status (0, non-smoker; 1, smoker) and energy intake. ^e^Mixed-effect models were applied for analyzing the associations between nutrient intake and LDL-C concentration with fixed effect: season (1, winter; 2, spring; 3 summer; 4, autumn), age, sex (0, men; 1, women), BMI, physical activity, ethanol consumption, smoking status (0, non-smoker; 1, smoker) and energy intake and random intercept: subject ID

No association with LDL-C concentration was observed for SNPs in the CC dataset (Table 3). In the OS and MI datasets of the cross-sectional study, negative relationships were shown for rs599839 and rs1160985, and a positive relationship was found for rs651007 (*P* < 0.05 for all). Likewise, in those datasets of the seasonal variation study, relationships to LDL-C concentration were shown for rs1160985 (negative) and rs651007 (positive) (*P* < 0.05 in each case). Overall, lower LDL-C concentrations were observed in summer.

**Table 3:** Associations between SNPs and LDL-C concentration in a cross-sectional study and a seasonal variation study

*Gene/season*	rs ID/season	CHR	Position	Alleles^a^	MAF	Genotype^b^			*P*_*HWE*_	Complete case dataset^c^			Observed subject dataset^d^			Multiple imputation dataset (m = 100)^e^		
						AA	Aa	aa		β	S.E.	*P*	β	S.E.	*P*	β	S.E.	*P*
Cross-sectional study^f^																		
Number of observations (person · season)											51			72			78	
*PSRC1*	rs599839	1	109279544	A>G	0.08	66	11	1	0.79	-7.1 × E-2	4.0 × E-2	0.08	-8.6 × E-2	3.5 × E-2	**0.01**	-8.0 × E-2	3.4 × E-2	**0.02**
*HMGCR*	rs12654264	5	75352778	T>A	0.44	25	38	15	1.00	3.5 × E-2	3.7 × E-2	0.35	2.1 × E-2	2.9 × E-2	0.47	2.3 × E-2	2.7 × E-2	0.40
*TIMD4*	rs6882076	5	156963286	C>T	0.15	56	21	1	0.82	-1.9 × E-2	3.7 × E-2	0.61	2.2 × E-2	2.8 × E-2	0.44	1.9 × E-2	2.7 × E-2	0.50
*TBL2*	rs17145738	7	73568544	C>T	0.11	62	15	1	1.00	-5.1 × E-2	3.6 × E-2	0.17	-5.5 × E-2	2.9 × E-2	0.06	-5.1 × E-2	2.9 × E-2	0.09
-	rs651007	9	133278431	C>T	0.28	37	39	2	0.08	5.6 × E-2	3.0 × E-2	0.06	4.8 × E-2	2.4 × E-2	0.05	4.8 × E-2	2.4 × E-2	**0.05**
*TOMM40*	rs1160985	19	44900155	C>T	0.35	32	38	8	0.80	-5.3 × E-2	3.2 × E-2	0.11	-6.4 × E-2	2.6 × E-2	**0.01**	-6.7 × E-2	2.5 × E-2	**0.009**
*APOC1*	rs445925	19	44912383	G>A	0.10	64	12	2	0.35	-2.0 × E-2	4.8 × E-2	0.68	-5.9 × E-2	3.3 × E-2	0.08	-5.8 × E-2	3.2 × E-2	0.07
Seasonal variation research^g^																		
Number of observations (person · season)											204			262			312	
*PSRC1*	rs599839									-4.8 × E-2	3.6 × E-2	0.19	-4.8 × E-2	3.1 × E-2	0.12	-4.7 × E-2	3.2 × E-2	0.15
Season	- Winter									Reference			Reference			Reference		
	- Spring									-1.5 × E-2	8.9 × E-3	0.11	-1.7 × E-2	7.8 × E-3	**0.03**	-1.6 × E-2	1.1 × E-2	0.13
	- Summer									-2.8 × E-2	8.9 × E-3	**0.002**	-2.4 × E-2	7.7 × E-3	**0.002**	-2.2 × E-2	1.1 × E-2	**0.04**
	- Autumn									-1.4 × E-2	9.0 × E-3	0.12	-1.5 × E-2	8.2 × E-3	0.08	-1.1 × E-2	1.1 × E-2	0.34
*HMGCR*	rs12654264									3.9 × E-2	3.3 × E-2	0.24	2.6 × E-2	2.4 × E-2	0.28	2.5 × E-2	2.6 × E-2	0.33
Season	- Winter									Reference			Reference			Reference		
	- Spring									-1.4 × E-2	8.9 × E-3	0.11	-1.7 × E-2	7.8 × E-3	**0.03**	-1.6 × E-2	1.1 × E-2	0.13
	- Summer									-2.8 × E-2	8.9 × E-3	**0.002**	-2.4 × E-2	7.7 × E-3	**0.002**	-2.2 × E-2	1.1 × E-2	**0.04**
	- Autumn									-1.4 × E-2	9.0 × E-3	0.12	-1.5 × E-2	8.2 × E-3	0.07	-1.1 × E-2	1.1 × E-2	0.34
*TIMD4*	rs6882076									6.5 × E-3	3.2 × E-2	0.84	1.8 × E-2	2.4 × E-2	0.45	1.9 × E-2	2.6 × E-2	0.47
Season	- Winter									Reference			Reference			Reference		
	- Spring									-1.4 × E-2	8.9 × E-3	0.11	-1.7 × E-2	7.8 × E-3	**0.03**	-1.6 × E-2	1.1 × E-2	0.13
	- Summer									-2.8 × E-2	8.9 × E-3	**0.002**	-2.4 × E-2	7.7 × E-3	**0.002**	-2.2 × E-2	1.1 × E-2	**0.04**
	- Autumn									-1.4 × E-2	9.0 × E-3	0.12	-1.5 × E-2	8.2 × E-3	0.07	-1.1 × E-2	1.1 × E-2	0.34
*TBL2*	rs17145738									-4.6 × E-2	3.2 × E-2	0.16	-4.8 × E-2	2.6 × E-2	0.08	-4.7 × E-2	2.8 × E-2	0.09
Season	- Winter									Reference			Reference			Reference		
	- Spring									-1.4 × E-2	8.9 × E-3	0.11	-1.7 × E-2	7.8 × E-3	**0.03**	-1.6 × E-2	1.1 × E-2	0.13
	- Summer									-2.8 × E-2	8.9 × E-3	**0.002**	-2.4 × E-2	7.7 × E-3	**0.002**	-2.2 × E-2	1.1 × E-2	**0.04**
	- Autumn									-1.4 × E-2	9.0 × E-3	0.12	-1.5 × E-2	8.2 × E-3	0.08	-1.1 × E-2	1.1 × E-2	0.34
-	rs651007									5.0 × E-2	2.7 × E-2	0.07	4.7 × E-2	2.1 × E-2	**0.03**	4.7 × E-2	2.3 × E-2	**0.04**
Season	- Winter									Reference			Reference			Reference		
	- Spring									-1.4 × E-2	9.0 × E-3	0.11	-1.7 × E-2	7.8 × E-3	**0.03**	-1.6 × E-2	1.1 × E-2	0.13
	- Summer									-2.8 × E-2	8.9 × E-3	**0.002**	-2.4 × E-2	7.7 × E-3	**0.002**	-2.2 × E-2	1.1 × E-2	**0.04**
	- Autumn									-1.4 × E-2	9.0 × E-3	0.11	-1.5 × E-2	8.2 × E-3	0.07	-1.1 × E-2	1.1 × E-2	0.34
*TOMM40*	rs1160985									-4.7 × E-2	2.8 × E-2	0.11	-5.9 × E-2	2.2 × E-2	**0.01**	-5.9 × E-2	2.4 × E-2	**0.01**
Season	- Winter									Reference			Reference			Reference		
	- Spring									-1.4 × E-2	9.0 × E-3	0.11	-1.6 × E-2	7.8 × E-3	0.04	-1.6 × E-2	1.1 × E-2	0.13
	- Summer									-2.8 × E-2	8.9 × E-3	**0.002**	-2.4 × E-2	7.8 × E-3	**0.002**	-2.2 × E-2	1.1 × E-2	**0.04**
	- Autumn									-1.4 × E-2	9.0 × E-3	0.12	-1.5 × E-2	8.2 × E-3	0.07	-1.1 × E-2	1.1 × E-2	0.33
*APOC1*	rs445925									-1.4 × E-2	4.3 × E-2	0.74	-4.9 × E-2	2.8 × E-2	0.08	-4.9 × E-2	3.0 × E-2	0.11
Season	- Winter									Reference			Reference			Reference		
	- Spring									-1.5 × E-2	8.9 × E-3	0.11	-1.7 × E-2	7.8 × E-3	**0.03**	-1.6 × E-2	1.1 × E-2	0.13
	- Summer									-2.8 × E-2	8.9 × E-3	**0.002**	-2.4 × E-2	7.7 × E-3	**0.002**	-2.2 × E-2	1.1 × E-2	**0.04**
	- Autumn									-1.4 × E-2	9.0 × E-3	0.12	-1.5 × E-2	8.2 × E-3	0.07	-1.1 × E-2	1.1 × E-2	0.34

*Abbreviations*: *SNP* single nucleotide polymorphism, *LDL-C* LDL cholesterol, *CHR* chromosome, *MAF* minor allele frequency, *PHWE P*-value for Hardy-Weinberg equilibrium, *m* number of imputations. The following variables were log-transformed to improve the normality: LDL-C concentration; BMI; physical activity; ethanol consumption. *P*-value <0.05 was shown as bold. ^a^Alleles were shown as following: major allele>minor allele. ^b^A major allele was shown as 'A', and a minor allele was shown 'a'. ^c^Complete case dataset was composed of subjects without missing values on four seasons. ^d^Observed subjects dataset was used for the analysis of all of values collected in each season even if there were missing values. ^e^Multiple imputation datasets were calculated by means of applying observed subjects dataset to the MI procedure. ^f^Multiple regression models were applied for analyzing the associations between SNPs and LDL-C concentration with adjustment for age, sex (0: men, 1: women), BMI, physical activity, ethanol consumption, smoking status (0: non-smoker, 1: smoker). ^g^Mixed-effect models were applied for analyzing the associations between SNPs and LDL-C concentration with age, sex (0: men, 1: women), BMI, physical activity, ethanol consumption, smoking status (0: non-smoker, 1: smoker) and season (1: winter, 2: spring, 3: summer, 4: autumn) as fixed effects and subject ID as random intercept

Table 4 shows the relationship between nutrient intake and SNPs on LDL-C concentration according to three dietary assessment methods in the two study designs. Results from three MI datasets are summarized as follows: A negative association was observed with rs1160985 (*P* = 0.007 and 0.03), considering that TDF intake derived from the FFQ and 4 s-3d WDRs was applied as a confounding factor. Similarly, a positive association was shown with rs651007 (*P* = 0.57 and 0.03). Finally, a negative relationship was observed with rs599839 (*P* = 0.03 and 0.07), taking into account dietary cholesterol intake derived from the WDR method, but not the FFQ. In the seasonal variation study, ethanol consumption and rs17145738 were negatively related (*P* < 0.05 for both). Most of nutrient intake was not related after adjusted for the effects of SNPs. Consistently, lower LDL-C concentrations were observed in summer (*P* < 0.05 for all).

**Table 4:** Associations among nutrient intake and SNPs on LDL-C concentration according to three dietary assessment methods

	Complete case dataset^a^			Observed subject dataset^b^			Multiple imputation dataset (m = 100)^c^		
	β	S.E.	*P*	β	S.E.	*P*	β	S.E.	*P*
Cross-sectional study									
FFQ (one year)^d^									
Number of observations (person · season)		51			73			78	
Cholesterol (mg/MJ)	4.5 × E-2	1.8 × E-1	0.81	2.4 × E-2	1.4 × E-1	0.87	3.3 × E-2	1.4 × E-1	0.81
*PSRC1 rs599839*	9.7 × E-2	5.0 × E-1	0.85	2.3 × E-2	4.6 × E-1	0.96	3.7 × E-2	4.5 × E-1	0.94
Interaction	-1.1 × E-1	3.4 × E-1	0.74	-7.0 × E-2	3.1 × E-1	0.82	-7.7 × E-2	3.1 × E-1	0.80
Cholesterol (mg/MJ)	1.5 × E-2	2.0 × E-1	0.94	-3.1 × E-2	1.6 × E-1	0.85	-2.4 × E-2	1.5 × E-1	0.87
*TIMD4 rs6882076*	-2.0 × E-1	4.6 × E-1	0.67	-8.1 × E-2	4.0 × E-1	0.84	-1.0 × E-1	4.0 × E-1	0.79
Interaction	1.2 × E-1	3.0 × E-1	0.70	7.0 × E-2	2.6 × E-1	0.79	8.1 × E-2	2.6 × E-1	0.75
TDF (g/MJ)	-1.7 × E-1	2.6 × E-1	0.53	-1.3 × E-1	2.3 × E-1	0.56	-8.4 × E-2	2.2 × E-1	0.70
- rs651007	3.5 × E-2	5.3 × E-2	0.51	1.5 × E-2	4.6 × E-2	0.75	2.5 × E-2	4.4 × E-2	0.57
Interaction	1.6 × E-1	2.9 × E-1	0.59	2.1 × E-1	2.6 × E-1	0.40	1.6 × E-1	2.5 × E-1	0.52
TDF (g/MJ)	-2.0 × E-1	2.1 × E-1	0.35	-9.8 × E-2	1.7 × E-1	0.57	-9.8 × E-2	1.7 × E-1	0.57
*TOMM40 rs1160985*	-1.1 × E-1	5.5 × E-2	0.05	-1.2 × E-1	4.4 × E-2	**0.008**	-1.2 × E-1	4.3 × E-2	**0.007**
Interaction	2.8 × E-1	2.8 × E-1	0.32	3.0 × E-1	2.3 × E-1	0.20	3.0 × E-1	2.3 × E-1	0.18
Ethanol (g/day)	-8.5 × E-2	2.9 × E-2	**0.006**	-3.7 × E-2	2.3 × E-2	0.11	-3.3 × E-2	2.3 × E-2	0.16
*TBL2 rs17145738*	-9.3 × E-2	5.1 × E-2	0.07	-5.4 × E-2	4.4 × E-2	0.23	-4.7 × E-2	4.5 × E-2	0.30
Interaction	3.1 × E-2	4.8 × E-2	0.52	-8.6 × E-3	4.3 × E-2	0.84	-1.3 × E-2	4.3 × E-2	0.77
First season 3-day WDRs^d^									
Number of observations (person · season)		51			72			78	
Cholesterol (mg/MJ)	1.7 × E-2	1.3 × E-1	0.90	-6.7 × E-2	9.2 × E-2	0.47	-6.0 × E-2	8.8 × E-2	0.50
*PSRC1 rs599839*	-1.1 × E+0	5.5 × E-1	**0.04**	-8.6 × E-1	3.8 × E-1	**0.03**	-8.4 × E-1	3.7 × E-1	**0.03**
Interaction	6.8 × E-1	3.5 × E-1	0.06	5.0 × E-1	2.4 × E-1	**0.04**	4.9 × E-1	2.4 × E-1	**0.05**
Cholesterol (mg/MJ)	1.3 × E-2	1.7 × E-1	0.94	-9.4 × E-3	1.1 × E-1	0.93	-5.4 × E-3	1.0 × E-1	0.96
*TIMD4* rs6882076	-2.3 × E-1	4.0 × E-1	0.57	2.5 × E-2	3.1 × E-1	0.94	6.0 × E-2	3.1 × E-1	0.85
Interaction	1.4 × E-1	2.6 × E-1	0.60	-3.4 × E-3	2.0 × E-1	0.99	-2.7 × E-2	2.0 × E-1	0.89
TDF (g/MJ)	-2.6 × E-1	1.4 × E-1	0.07	-1.9 × E-1	1.3 × E-1	0.14	-1.4 × E-1	1.3 × E-1	0.26
- rs651007	6.8 × E-2	5.6 × E-2	0.23	1.1 × E-2	4.5 × E-2	0.82	2.3 × E-2	4.5 × E-2	0.61
Interaction	-7.6 × E-2	2.0 × E-1	0.71	1.5 × E-1	1.7 × E-1	0.37	9.6 × E-2	1.7 × E-1	0.56
TDF (g/MJ)	-2.7 × E-1	2.0 × E-1	0.19	-7.1 × E-2	1.3 × E-1	0.58	-6.9 × E-2	1.3 × E-1	0.58
*TOMM40* rs1160985	-3.9 × E-2	6.4 × E-2	0.55	-4.8 × E-2	4.7 × E-2	0.32	-6.1 × E-2	4.6 × E-2	0.19
Interaction	-1.1 × E-2	2.2 × E-1	0.96	-3.3 × E-2	1.7 × E-1	0.84	-1.0 × E-2	1.6 × E-1	0.95
Ethanol (g/day)	-7.7 × E-2	3.4 × E-2	**0.03**	-6.3 × E-2	2.5 × E-2	**0.02**	-5.1 × E-2	2.5 × E-2	**0.05**
TBL2 rs17145738	-6.6 × E-2	5.5 × E-2	0.24	-6.8 × E-2	4.7 × E-2	0.15	-6.3 × E-2	4.6 × E-2	0.18
Interaction	2.5 × E-2	7.0 × E-2	0.72	2.2 × E-2	6.4 × E-2	0.73	1.6 × E-2	6.3 × E-2	0.80
Seasonal variation research									
Four season 3-day WDRs^e^									
Number of observations (person · season)		204			262			312	
Cholesterol (mg/MJ)	5.6 × E-2	3.2 × E-2	0.08	3.7 × E-2	2.7 × E-2	0.18	3.2 × E-2	4.3 × E-2	0.46
*PSRC1* rs599839	-2.9 × E-1	1.5 × E-1	**0.05**	-2.9 × E-1	1.3 × E-1	**0.02**	-2.9 × E-1	1.6 × E-1	0.07
Interaction	1.5 × E-1	8.9 × E-2	0.10	1.5 × E-1	7.7 × E-2	0.05	1.5 × E-1	1.0 × E-1	0.12
Season - Winter	Reference			Reference			Reference		
- Spring	-1.8 × E-2	9.0 × E-3	**0.05**	-1.9 × E-2	7.7 × E-3	**0.01**	-2.0 × E-2	1.1 × E-2	0.06
- Summer	-2.9 × E-2	9.0 × E-3	**0.001**	-2.6 × E-2	7.7 × E-3	**<0.001**	-2.5 × E-2	1.1 × E-2	**0.02**
- Autumn	-1.9 × E-2	9.0 × E-3	**0.04**	-1.8 × E-2	8.2 × E-3	**0.03**	-1.5 × E-2	1.1 × E-2	0.18
Cholesterol (mg/MJ)	3.8 × E-2	3.6 × E-2	0.29	2.9 × E-2	3.0 × E-2	0.34	2.4 × E-2	4.7 × E-2	0.60
*TIMD4* rs6882076	-1.8 × E-1	1.1 × E-1	0.09	-1.3 × E-1	9.4 × E-2	0.18	-9.9 × E-2	1.2 × E-1	0.42
Interaction	1.2 × E-1	6.6 × E-2	0.07	9.2 × E-2	5.8 × E-2	0.12	7.5 × E-2	7.7 × E-2	0.33
Season - winter	Reference			Reference			Reference		
- spring	-1.7 × E-2	9.0 × E-3	0.07	-1.8 × E-2	7.7 × E-3	**0.02**	-1.9 × E-2	1.0 × E-2	0.08
- summer	-3.1 × E-2	9.0 × E-3	**<0.001**	-2.7 × E-2	7.7 × E-3	**<0.001**	-2.5 × E-2	1.1 × E-2	**0.02**
- autumn	-1.9 × E-2	9.0 × E-3	**0.04**	-1.8 × E-2	8.2 × E-3	**0.03**	-1.5 × E-2	1.1 × E-2	0.19
TDF (g/MJ)	3.1 × E-2	4.2 × E-2	0.46	1.8 × E-2	3.9 × E-2	0.64	7.0 × E-3	5.4 × E-2	0.90
- rs651007	7.3 × E-2	3.1 × E-2	**0.02**	6.8 × E-2	2.5 × E-2	**0.007**	6.3 × E-2	2.8 × E-2	**0.03**
Interaction	-9.4 × E-2	6.5 × E-2	0.15	-8.9 × E-2	5.7 × E-2	0.12	-6.3 × E-2	7.5 × E-2	0.40
Season - winter	Reference			Reference			Reference		
- spring	-1.5 × E-2	9.1 × E-3	0.10	-1.7 × E-2	7.8 × E-3	**0.03**	-1.8 × E-2	1.1 × E-2	0.09
- summer	-2.9 × E-2	9.2 × E-3	**0.002**	-2.6 × E-2	7.9 × E-3	**<0.001**	-2.4 × E-2	1.1 × E-2	**0.03**
- autumn	-1.5 × E-2	9.0 × E-3	0.11	-1.5 × E-2	8.2 × E-3	0.07	-1.2 × E-2	1.1 × E-2	0.27
TDF (g/MJ)	-4.7 × E-2	6.6 × E-2	0.47	-7.5 × E-2	5.4 × E-2	0.17	-5.5 × E-2	8.0 × E-2	0.49
*TOMM40* rs1160985	-6.0 × E-2	3.3 × E-2	0.07	-7.5 × E-2	2.6 × E-2	**0.005**	-7.0 × E-2	3.1 × E-2	**0.03**
Interaction	5.8 × E-2	7.5 × E-2	0.44	8.1 × E-2	6.3 × E-2	0.20	5.8 × E-2	8.7 × E-2	0.50
Season - winter	Reference			Reference			Reference		
- spring	-1.6 × E-2	9.4 × E-3	0.10	-1.8 × E-2	7.9 × E-3	**0.03**	-1.8 × E-2	1.1 × E-2	0.09
- summer	-2.9 × E-2	9.4 × E-3	**0.002**	-2.6 × E-2	7.9 × E-3	**0.001**	-2.4 × E-2	1.1 × E-2	**0.03**
- autumn	-1.5 × E-2	9.2 × E-3	0.10	-1.6 × E-2	8.3 × E-3	0.06	-1.3 × E-2	1.1 × E-2	0.24
Ethanol (g/day)	-3.0 × E-2	1.5 × E-2	**0.05**	-2.9 × E-2	1.2 × E-2	**0.01**	-3.6 × E-2	1.7 × E-2	**0.03**
*TBL2* rs17145738	-7.0 × E-2	3.7 × E-2	0.06	-7.3 × E-2	2.9 × E-2	**0.02**	-6.9 × E-2	3.3 × E-2	**0.04**
Interaction	4.3 × E-2	3.0 × E-2	0.15	4.7 × E-2	2.4 × E-2	0.05	4.2 × E-2	3.3 × E-2	0.21
Season - winter	Reference			Reference			Reference		
- spring	-1.3 × E-2	8.9 × E-3	0.15	-1.5 × E-2	7.7 × E-3	0.05	-1.6 × E-2	1.1 × E-2	0.13
- summer	-2.9 × E-2	8.9 × E-3	**0.001**	-2.4 × E-2	7.7 × E-3	**0.002**	-2.3 × E-2	1.1 × E-2	**0.03**
- autumn	-1.4 × E-2	8.9 × E-3	0.11	-1.5 × E-2	8.1 × E-3	0.07	-1.2 × E-2	1.1 × E-2	0.27

*Abbreviations*: *SNP* single nucleotide polymorphism, *LDL-C* LDL cholesterol, *FFQ* food frequency questionnaire, *TDF* total dietary fiber, *3-day WDRs* 3-day weighed dietary records, *m* number of imputations. The following variables were log-transformed to improve the normality: LDL-C concentration; dietary cholesterol; TDF; ethanol consumption; BMI; physical activity. *P* <0.05 was shown as bold. ^a^Complete case dataset was composed of subjects without missing values on four seasons. ^b^Observed subjects dataset included all subjects from whom data were collected in each season for analysis, even if there were some missing values. ^c^Multiple imputation datasets were calculated by means of applying the MI procedure to the observed subjects dataset. ^d^Multiple regression models were applied for the analysis of the association among nutrient intake and SNPs on LDL-C concentration with adjustments for age, sex (0, men; 1, women), BMI, physical activity, ethanol consumption, smoking status (0, non-smoker; 1, smoker) and energy intake. ^e^Mixed-effect models were applied for the analysis of the association among nutrient intake and SNPs on LDL-C concentration with fixed effect: season (1, winter; 2, spring; 3 summer; 4, autumn), age, sex (0, men; 1, women), BMI, physical activity, ethanol consumption, smoking status (0, non-smoker; 1, smoker) and energy intake and random intercept: subject ID.

## Discussion

LDL-C concentration was negatively associated with intake of TDF derived from the first season 3-day WDRs in the CC dataset of the cross-sectional study. It was positively associated with dietary cholesterol intake in both the CC and OS datasets in the seasonal variation study. These effects were diminished in the MI dataset. In contrast to these effects, ethanol consumption was clearly related to LDL-C concentration in all three datasets and dietary assessment methods. Using mixed-effect models, seasonal effects of summer were observed on both diet and LDL-C concentration. In the MI dataset of the seasonal variation study, a negative relationship to LDL-C concentration was shown each ethanol consumption and rs17145738. LDL-C concentration was related to rs1160985 (negatively) and rs651007 (positively), taking into account TDF intake as a confounding factor. It was negatively associated with rs599839 after adjusting for dietary cholesterol intake.

Using a multiple regression analysis method, our FFQ was scientifically developed to detect inter-individual differences, and validated these with reference to four season 7-day WDRs [10, 20, 21]. For LDL-C concentration, negative associations with ethanol consumption were consistently observed, independent of dietary assessment methods, seasonal effects and missing values. It was thought that alcohol consumption was modified by seasonal changes in consumption, but not remarkably affected by recall bias. Looking at all three datasets, a significant negative association with TDF intake on LDL-C concentration was not consistently observed. Unlike a multiple regression model, a mixed-effect model was maximally able to be applied all of the collected data, which included not only objective and explanatory variables, but also covariates, against missing values. Then fixed and random effects, i.e., season and subjects, were distinctively adjusted on the associations with LDL-C concentration in the CC and OS datasets. In the CC dataset, selection bias should be noted for the subjects in order to interpret the associations, as their characteristics might not be reflected in a target population. In the OS dataset with a mixed-effect model, selection bias was attenuated; selection bias is minimalized in the MI dataset. Therefore, a recent systematic review recommended that MI is used in these types of analyses, reducing inappropriately discussions about selection bias and inaccurate information derived from data with missing values [17]. The procedures used in MI should be appropriately handled step by step [16].

Ultimately, in the MI dataset of seasonal variation study, relationships between dietary and genetic factors for LDL-C concentration can be summarized as follows. First, although ethanol consumption and rs17145738 was negatively associated, the role of rs17145738 in lowering LDL-C concentration has been not clarified. However, a Chinese population study showed negative relationships between LDL-C concentration and rs17145738 or alcohol consumption according to ethnic group and gender, and a consistent positive relationship between HDL cholesterol concentration and alcohol consumption but not rs17145738 only in the Han male population [40]. Among lipoproteins, the effects of moderate ethanol consumption especially on LDL-C concentration has been widely discussed [6]. Second, most of dietary factors were shown to mask genetic functions. Third, unlike other dietary factors, TDF intake was not masked the genetic functions of rs651007 and rs1160985 on LDL-C concentration, and was instead a confounding factor. The SNP of rs651007 (which has shown linkage disequilibrium with rs579459, as shown in “Methods”) is near the 5′ end of the ABO gene, which codes for the ABO blood group and soluble P- and E-selectin (soluble intercellular adhesion molecule-1) related to LDL-C concentration [41, 42], and rs1160985 was related to LDL-C concentration through the gut microbiota [43, 44].

Fourth, cholesterol intake was also not masked the genetic function of rs599839 on LDL-C concentration. Fifth, two interactions were observed in the OS dataset, and a marginal one between dietary cholesterol intake and rs599839, which is located in the intergenic region between *PSRC1* and *SORT1,* was remained; higher level of SORT1 mRNA has been known to be associated with higher uptake of LDL into cells [45, 46]. Sixth, in comparison to winter, seasonal differences in effects were observed in summer. This finding was consistent with previous large-scale, cross-sectional studies (e.g., 0.11 to 0.18 mmol/L difference in LDL-C concentration between winter and summer [4, 5], and this was thought to be related to seasonal variation in nutrient intake, physical activity, plasma volume haemoconcentration in winter and haemodilution in summer) [47]. One of the strong points of the current study was that we collected four samples from the same subjects in each season and subsequently showed the seasonal effects according to the dietary intakes of nutrients (which were derived from each of the three dietary assessment methods) and SNPs related to LDL-C concentration, after adjusting for ordinal confounding factors such as age, sex, BMI, and lifestyle.

The current study has several limitations. First, study subjects were not randomly selected from the general population, but were representative of people living in a defined area. Second, the sample size was inadequate for assessing relationships between dietary and genetic factors. However, dietary assessment methods were able to be successfully executed according to the FFQ and the first and four season 3-day WDRs among the same subjects with a large number of SNPs. Third, there was a significant number of missing values in the seasonal variation study because it was difficult to collect both a 3-day WDR and blood samples in every seasons from the same subjects. Fourth, the SNPs in our study were not selected by GWAS. If the systematic literature review which was conducted to select the SNPs for our analysis was characterized as the discovery phase, the current study can be thought of as the replication phase.

## Conclusions

In conclusion, we were able to identify specific relationships between dietary and genetic factors on LDL-C concentration, according to multiple comparisons from two study designs, three dietary assessment methods and seasonal variation. Additionally, we utilized MI for handling missing values. Unlike nutrient intake, ethanol consumption derived from three dietary assessment methods was consistently related to LDL-C concentration. Critical relationships with LDL-C concentration were demonstrated for some SNPs after adjusting for specific nutrients derived from the WDRs, taking into account seasonal effects. Our results have implications for public health and clinical practice on the prevention of dyslipidemia. It can be used to help to interpret the relationships between dietary and genetic factors on LDL-C concentration in large-scale epidemiological studies using FFQs.

## Supplementary information

**Additional file 1.** Genome-wide association studies for LDL cholesterol concentration among East Asians (Japanese, Chinese, Korean). Abbreviations: SNPs, single nucleotide polymorphisms; CHR, chromosome; MAF, minor allele frequency. ^a^Alleles were presented as major/minor or reference/alternative. ^b^Effect sizes are shown as β ± standard error (SE) or β [95% confidence interval (CI)].

## Data Availability

The datasets generated and/or analyzed during the current study are not publicly available due to maintenance of the participants’ privacy but are available from the corresponding author on reasonable request.

## Abbreviations

4 s-3d WDRs: Four season 3-day weighed dietary records
BMI: Body mass index
CC: Complete case
CVD: Cardiovascular disease
FFQ: Food frequency questionnaire
GWAS: Genome-wide association study
LDL-C: LDL cholesterol
MI: Multiple imputation
OS: Observed subject
SNP: Single nucleotide polymorphism
TDF: Total dietary fiber
WDR: Weighed dietary record
